# Personality and social environment predict cognitive performance in common marmosets (*Callithrix jacchus*)

**DOI:** 10.1038/s41598-022-10296-8

**Published:** 2022-05-05

**Authors:** Vedrana Šlipogor, Christina Graf, Jorg J. M. Massen, Thomas Bugnyar

**Affiliations:** 1 Department of Behavioral and Cognitive Biology, University of Vienna, Vienna, Austria; 2 Department of Zoology, Faculty of Science, University of South Bohemia, Branišovská 1760, 37005 České Budějovice, Czech Republic; 3 Animal Behaviour and Cognition Group, Institute of Environmental Biology, Utrecht University, Utrecht, The Netherlands

**Keywords:** Animal behaviour, Biological anthropology

## Abstract

Consistent inter-individual variation in cognition has been increasingly explored in recent years in terms of its patterns, causes and consequences. One of its possible causes are consistent inter-individual differences in behaviour, also referred to as animal personalities, which are shaped by both the physical and the social environment. The latter is particularly relevant for group-living species like common marmosets (*Callithrix jacchus*), apt learners that display substantial variation in both their personality and cognitive performance, yet no study to date has interlinked these with marmosets’ social environment. Here we investigated (i) consistency of learning speed, and (ii) whether the PCA-derived personality traits Exploration-Avoidance and Boldness-Shyness as well as the social environment (i.e., family group membership) are linked with marmosets’ speed of learning. We tested 22 individuals in series of personality and learning-focused cognitive tests, including simple motor tasks and discrimination learning tasks. We found that these marmosets showed significant inter-individual consistency in learning across the different tasks, and that females learned faster than males. Further, bolder individuals, and particularly those belonging to certain family groups, learned faster. These findings indicate that both personality and social environment affect learning speed in marmosets and could be important factors driving individual variation in cognition.

## Introduction

Cognition is broadly defined as gathering, processing, storing and using information^1^, and it includes a wide array of capabilities, from attention and categorization to individual and social learning, self-recognition and language^2^. In non-human animals, cognitive abilities are often explored in terms of survival and fitness^3,4^, and typically linked to challenges faced in social life^5,6^ and/or during foraging^7–9^. Yet, the required biological hardware is costly, which leads to trade-offs, such as those between longevity and brain to body masses^10^, which can explain a lot of the variation in cognitive abilities between species^11^. Causes of intra-specific variation in animal cognitive performance were, however, largely overlooked in the past, and in particular, the non-performers were excluded or treated as outliers^11,12^. This is surprising, considering that consistent differences in human cognitive capabilities have been well-established, and are considered as a major source of phenotypic plasticity^12,13^.

To better understand the patterns of intra-specific variation in cognition and to assess consistency in performance, cognitive test batteries have been developed^3,12,14–16^. These usually include an array of different cognitive tests that measure individuals’ performance in, for instance, associative learning tasks, i.e., tasks where subjects associate an *object* with a reward; operant learning tasks where subjects learn to associate an *action* with a reward, and discrimination learning tasks, where subjects learn to distinguish between two objects, one leading to a reward (S+), and the other one not (S−); or reversal learning tasks that measure how a subject’s behaviour changes according to a change in the environment, i.e., whether subjects are successful in inhibiting behaviour that has previously been successful (for other examples, please see^12^). Researchers often look at the speed of learning, trials to criterion or accuracy of learning to quantify cognitive performance^12^. A recent meta-analysis^17^ confirmed that cognitive performance shows considerable temporal and contextual consistency, with repeatability estimates ranging between 0.15 and 0.28 across studies. Further, it seems that sex explains a considerable amount of variation in effect sizes in the speed of learning^18^, possibly due to males and females having different selective pressures on cognitive abilities (that are reflected in fitness^19^), and/or different motivation levels to perform in cognitive tasks (e.g.^20^). Nevertheless, considerable variation remains even within the sexes, which may be mediated by more general inter-individual differences in behaviour^21–23^.

Inter-individual behavioural differences that are consistent over time and/or across different contexts are typically referred to as animal personalities^24–26^. Like the human “Big Five” personality constructs (i.e. Openness to Experience, Conscientiousness, Extraversion, Agreeableness and Neuroticism^27^), animal personalities are often described by the five broad personality constructs, i.e., Boldness-Shyness, Exploration-Avoidance, Activity, Aggressiveness and Sociability^25^. Yet, the combinations of these, and potentially other constructs, that all face some problems, like that of labelling and the jingle-jangle fallacy^28^, depend on the species’ socio-ecology^29^. Personality constructs can have adaptive value^3,30,31^, tend to be independent of an animal’s sex, age or size (^25^; but see e.g.,^32^), and may be modified based on the social environment^33,34^. Especially in species living in structured social groups, social partners may be similar in personality^35–38^ and/or group members may have more similar personality traits than members of other groups^39–42^.

Individual differences in learning and their link to certain personality traits have been well-established in human personality research (see review^43^). For example, high Conscientiousness, Openness and Agreeableness scores have been linked with high school and academic performance (as measured with Grand Point Average, i.e., GPA) in humans, so it seems that not only being diligent, but also being curious and cooperative with others might drive individual learning success^44,45^. Research on non-human animals tends to support a link between individual learning or innovativeness and personality traits^15,46–48^, yet the direction of the relationship varies between studies and species (see a recent meta-analysis^18^). Personality traits like proactivity, exploration or boldness are usually associated with resource acquisition or risky behaviours and determine the speed and amount of exploration of novel environments, food, or objects^14,22,49^. Accordingly, very explorative animals (or those scoring high on the factor ‘Openness’) learn faster than the less explorative or less open ones (e.g., touch screen: chimpanzees, *Pan troglodytes*^50^, reversal learning: common pheasants, *Phasianus colchicus*, and great tits, *Parus major*^3,51^, discrimination learning: mice, *Mus musculus,* and male starlings, *Sturnus vulgaris*^48,52^), but they are also less accurate than the less explorative animals (discrimination learning: black-capped chickadees, *Poecile atricapillus*^53^). Further, bolder and more active animals tend to reach the set criterion in associative or spatial learning tasks sooner than shy and less active animals (bank voles, *Myodes glareolus,* and Eastern water skinks, *Eulamprus quoyii*^54,55^), but they do not seem to adjust quickly in reversal learning tasks^15,54^.

In contrast to these patterns, some studies did not find any of the predicted links between personality type and cognitive performance^22^. For instance, exploration, activity and neophilia were not linked with measures of cognitive performance in wild grey mouse lemurs, *Microcebus murinus*^56^, and domestic goats, *Capra hircus*, scoring lower on exploration, performed better than more explorative goats in a non-associative task (^57^; see other examples in^18^). Note that absence of a link between personality and cognitive tasks, or a lack of consistency across several cognitive tasks, might be due to different demands for these tasks, but also due to non-cognitive factors like motivation, hunger, breeding status, environmental conditions, or previous experience (cf.^11^). Thus, it is important to ensure that all individuals have standardized testing conditions and/or to account for the possible confounding effects of non-cognitive factors^12,58^.

Another well-established factor affecting personality and learning is the environment, both physical and social. In terms of the physical environment, some recent studies show that levels of boldness, exploration and behavioural flexibility of animals found in challenging urbanized habitats are higher than those of animals living in rural environments (striped field mice, *Apodemus agrarius*^59^, common voles, *Microtus arvalis*^60^, Eastern grey squirrels, *Sciurus carolinensis*^61^, Eurasian red squirrels, *Sciurus vulgaris*^62^). The social environment of animals is often determined by group size, composition, and relationships (e.g.,^63–65^). However, populations of the same species may differ in these attributes, which may subsequently influence the link between personality and cognitive performance (cf.^66^). For example, in pond snails (*Lymnaea stagnalis*), the link between exploratory behaviour and memory varied across solitary and social settings, as well as between laboratory and wild populations, suggesting that links between personality and cognition may not be consistent across different social contexts or physical environments^66^. Such an effect might be profound in highly social or cooperatively breeding species, that show particularly high levels of behavioural synchrony, cooperation and prosociality to their social groups^67–69^. In these species even subtle differences in social environment (i.e., *between* groups) may be reflected as differences in cognitive performance. Further, *within* social groups different personality types might adopt distinct problem-solving strategies, for example, in the context of group foraging, different individuals may turn to either social learning or scrounging (e.g.,^70–73^).

In this study we focus on common marmosets (*Callithrix jacchus*), cooperatively breeding New World primates that live in a variety of different habitats^74^. As is typical for Callithrichids, marmosets form cohesive family groups^75,76^ usually consisting of a single breeding pair (i.e., “breeders”) and non-breeding offspring (i.e., “helpers”), although the groups may somewhat vary in their social structure^77^. Callithrichids have already been shown to exhibit differences on a genus level in their neophilia and innovation^78^, but whether this variation also exists on the species level has not been studied yet. Common marmosets readily participate in behavioural and cognitive experiments under laboratory and field conditions^79^. Previous studies revealed their substantial learning and memory skills^70,80–83^ and found large inter-individual differences in individual and social learning tasks (e.g.,^84–86^). Moreover, common marmosets display consistent inter-individual differences in a battery of personality tests^40–42,87^, when assessed via behavioural observations^88–90^ or via questionnaires^91–93^, and when studied with a combination of different personality methods^89,94^. However, no study to date investigated whether both consistent inter-individual behavioural variation and the social environment affect marmosets’ propensity for learning.

Our aims were to see: (i) whether marmosets show consistency in learning speed across different tasks, (ii) whether personality affects the speed of individual learning, and if this is dependent on marmosets’ social environment (i.e., family group membership). We expected marmosets to show inter-individual differences in learning and that, in accordance with previous findings (e.g.,^81,95^), marmosets would not differ in terms of their sex, age or breeding status in how fast they learn to associate a certain action or a choice in a task with a reward. However, we expected learning speed to be affected by personality; in particular, in line with the speed-accuracy trade-off model^15,22^, we expected that bolder and/or more explorative individuals would learn faster. As three previous studies independently found marmoset personalities to be more similar within than between social groups^40–42^, we furthermore speculated that the strength of the effect of personality on learning speed might also depend on group membership.

## Methods

### Subjects and animal care

The common marmosets’ housing conditions at the Department of Behavioral and Cognitive Biology, Faculty of Life Sciences, University of Vienna, Austria, were in accordance with the Austrian legislation and the European Association of Zoos and Aquaria (EAZA) husbandry guidelines for Callitrichidae (see Electronic Supplementary Material [ESM] for details). Personality results are based on a sample of 27 individuals, learning tasks on 25 individuals, and 22 of individuals participated in both personality and learning tasks. This discrepancy in task participation was due to their natural life cycles: some were either not born yet when the personality tests took place, or they died before we conducted the cognitive tasks. We did not exclude any individual from testing for non-participation.

### Ethical statement

We followed all applicable international, national, and institutional guidelines for the care and use of animals. The study was approved by the Animal Ethics and Experimentation Board, Faculty of Life Sciences, University of Vienna (license number 2015–013), adhered to the legal requirements of Austria, to the American Society of Primatologists’ principles for the ethical treatment of primates and complied with the ARRIVE guidelines^96^.

### Behavioural tests: cognitive performance

We tested 25 common marmosets (16 males and 9 females, between 0.5 and 15 years old; Mdn = 8) from 5 different family groups, in two categories of cognitive tasks: simple motor tasks (SMT), designed to test the association between a motor action or between an object and a food reward, and discrimination learning tasks (DLT), designed to test the association between a rewarded and a non-rewarded object. The SMTs consisted of three tasks: (i) training subjects to hold a target, i.e., a small ball-point at the top of an expandable clicker stick (‘Target’, T; Supplementary Fig. S1), (ii) habituating subjects to a test room with help of a target (‘Room’, R; Supplementary Fig. S2), and (iii) training subjects to remain on a scale while holding a target (‘Scale’, S; Supplementary Fig. S3). The DLTs consisted of two tasks: (i) distinguishing between two objects of the same size, but with different features (‘Discrimination Feature’, DF; Supplementary Fig. S4a), and (ii) differentiating between two identical objects of different size (‘Discrimination Size’, DS; Supplementary Fig. S4b). In all tasks, we set elaborate training criteria and measured the time (in seconds) subjects needed to reach the criterion (see ESM for details, Supplementary Tables S1–S3). We used a time variable to quantify performance in all tasks because otherwise it would not be possible to compare performance across different tasks (e.g., in SMTs, we could not quantify errors). We used exclusively positive reinforcement methods in all tasks. In SMTs we used a so-called ‘clicker training’, a process in which a primary reinforcer (a food reward) is paired with a conditioned reinforcer (a hand-held clicker), which then becomes a reward for the desired behavioural outcomes^97^. The target stick was an already familiar object to the subjects from the keeping rooms, and standing on different types of scales was also a familiar action to subjects, as scales were used in regular weighing procedures. We used both ‘Target’ and ‘Scale’ task as they measure slightly different capabilities: the ‘Target’ task does not require marmosets to stay in one place for an extended amount of time (approximately 3 s), whereas the ‘Scale’ task does, and for these monkeys, staying in one place is more difficult than being able to move around.

### Cognitive tests: experimental procedure

Most monkeys were already familiar with the small experimental cage and the passageway tunnel system in the keeping rooms from previous studies and regular husbandry procedures, yet they got additionally habituated to these, the experimenter, and the experimental routine, to minimize any possible effect of stress during testing and to maximize positive association between the subjects and the experimenters during experimenter habituation and in pre-sessions described below. Most tasks were conducted by one experimenter (CG), whereas ‘Discrimination Size’ was conducted by another female student trained by CG to maintain consistency in the testing procedure. We used two different experimental cages depending on the task. ‘Target’, ‘Scale’ and both DLTs (i.e., ‘Discrimination Feature’, ‘Discrimination Size’) were conducted in a small experimental cage where also the personality tests took place (length × width × height; 152 cm × 42 cm × 110 cm), connected to the indoor home enclosures with a passageway system of tunnels. The subjects were visually isolated during the experiments. ‘Room’ task was conducted in a bigger test cage (length x width x height; 300 cm × 100 cm × 200 cm; see Supplementary Fig. S2), located in a separate test room, accessible through a passageway tunnel system leading through hallway to the home enclosures. Thus, it was isolated visually, auditory, and olfactory from the home enclosures. We took care that the gaps between two testing days for every individual were kept as short as possible (i.e., mostly there was a 0–1 day gap between two testing days). The longest gap between two sessions in SMTs was 12 days within a ‘Target’ task for subjects Sparrow, Kobold and Smart (due to management reasons), and in DLTs was 7 days within a ‘Discrimination Feature’ task for subject Locri. Prior to the first phase of all SMT tasks, to reduce possible neophobia, subjects received a single pre-session in a group setting. The monkeys were then trained individually in all tasks and phases apart from the ‘Room’ and ‘Scale’. In ‘Room’, the monkeys were trained together with their group in the first four phases of the task, and individually in the final phase of the task. In ‘Scale’, the monkeys were trained in their group in the first phase of the task and individually in the other two phases of the task. Every task (‘Target’, ‘Room’, ‘Scale’, ‘Discrimination Feature’, ‘Discrimination Size’) was divided in different phases where predetermined training goals were set, and rewards were adjusted accordingly, depending on the task and phase (see ESM, Supplementary Tables S1–S3). Subjects that did not reach the training goal, received a maximum latency for the trial/session/task. The order of the cognitive tasks was constant for all subjects, namely ‘Target’- ‘Room’- ‘Scale’- ‘Discrimination Feature’- ‘Discrimination Size’, except for one group (‘Sparrow’) for which the order of ‘Target’ and ‘Room’ tasks was reversed due to facility management.

### Cognitive tests: simple motor tasks

The goal of the ‘Target’ task was to train subjects to the principle of clicker training, namely to (i) associate the clicker with the food reward, (ii) touch the target with one or both hands, and (iii) hold the target for an extended period (approx. 3 s). The monkeys had to successfully complete three phases á five sessions, in individual setting, to reach the overall goal of ‘Target’ task, i.e. to hold the target for three seconds. One session lasted for a maximum of five minutes or once 10 rewards were given to the subject (i.e., cooked green beans or pieces of rice waffles, based on individual preferences; all subjects but Ginevra preferred green beans). Every subject participated in one session per day. For further details, see Supplementary Table S1 and Supplementary Figure S1.

The goal of the ‘Room’ task was to habituate subjects to a test cage located in the test room. The subjects were trained in a stepwise procedure consisting of five phases, with a help of the target, to use the passageway hallway system connecting their home enclosure to the new experimental room, to reach the overall training goal, i.e., to use all parts of the test cage when separated from the group. In the first four phases á two sessions for a maximum of five minutes, the subjects had increasingly more access to the test room and cage. In the final phase á five sessions of maximum five minutes, the subjects were trained individually, while their family group was in an adjoining compartment. Every subject participated in at most one training session per day. See Supplementary Table S2 and Supplementary Figure S2 for further details. One drawback of this test is that it may be convoluted with personality traits indicating bold or explorative tendencies of individuals. Even though in this test, unlike in standard personality tests, individuals are rewarded with food for a desired behavioural outcome, their personality traits may nevertheless further differ and influence outcome of the test.

The goal of the ‘Scale’ task was to train all subjects to remain on a weighing scale (infant scale MBSC Ultra U-2; weight resolution 2 g) for approximately 3 s while holding a target with one or both hands, and it was built on the ‘Target’ task. Phase 1 was conducted in a group setting and was completed after two sessions that lasted for five minutes, without an upper reward limit (i.e., number of green beans). In phases 2 and 3 the subjects were trained individually in five consecutive sessions. Every subject participated in one session per day which lasted for a maximum of five minutes or when 10 rewards were given. For details, see Supplementary Table S3 and Supplementary Figure S3.

### Cognitive tests: discrimination learning tasks

In DLTs, the subjects had to reliably distinguish between two different objects, and thus to learn to associate a particular object with a reward (hereafter, S+). In ‘Discrimination Feature’ task, the subjects had to distinguish between two objects of the same size, but different colour and shape: a blue rubber rabbit toy (length × width, 7 cm × 5 cm), and a yellow rubber duck toy (length x width, 5 cm × 7 cm) (Supplementary Fig. S4a). In ‘Discrimination Size’ task, the subjects had to distinguish between two objects with the same colour and shape, but different size: a big plastic ball (diameter = 10 cm), and a small plastic ball with black and white pattern (diameter = 4 cm) (Supplementary Fig. S4b).

All subjects were first tested in DF and then in DS task. The (S+) objects were assigned in a counterbalanced manner, that is, within each family group half of the subjects were trained for one object and the other half for the other object, and we also took care to counterbalance for sex and age. The testing order in a day was semi-randomized. To pre-habituate subjects to the boards and the objects and to avoid possible neophobic reactions, the objects were placed in front of the subjects’ home cages for five days before the tests started. We used banana or rice waffle pieces as rewards, depending on the previously established individual preferences. In both tasks, the objects were mounted on wooden boards (length x width; 40 × 20 cm), 20 cm apart, and the boards were placed on the outside of the small experimental cage. Subjects touched the objects through the wire mesh with one or both hands. The placement of the objects on the boards within the sessions was randomized; with a maximum of two consecutive trials with the same placement. Prior to start of ‘Discrimination Feature’ and ‘Discrimination Size’ task, one session was used to ‘bias’ the monkeys for their (S+) object, that is, a piece of banana was placed on the (S+) object for a total of 16 trials, to control for any possible pre-existing individual preferences of objects. The trials started once the wooden board with objects was placed in front of the experimental cage and ended after a maximum of 60 s. After the subjects touched one of the objects, the trial ended and the wooden board with objects was removed. Touching the predetermined (S+) object with one or both hands was counted as a ‘positive’ choice and was rewarded, whereas touching (S−) was counted as ‘negative’ choice and was not rewarded. Touching none of the objects was counted as ‘no choice’, and a piece of food was placed between the two objects to encourage further participation. If the subject took the food, the board was removed from view for 20 s, and then the same trial was repeated as a ‘second chance’ trial. Within one session a maximum of three ‘second chance’ trials were allowed. If the food in the middle was still ignored, the trial was counted as ‘no choice’. We video-recorded all trials. Additionally, we kept written notes on individuals’ progress. We video-coded the total time in seconds that an individual needed to reach criterion. In three individual sessions, due to technical issues with video camera, we replaced video-coded behaviour with written notes (DS; Aurora: session from 04.07.2017, Vento: session from 29.04.2017, Fimo: sessions from 29.04.2017 and 05.05.2017). One session consisted of 16 trials. We set the training criterion to 80% correct choices over three consecutive sessions (i.e., the subjects had to make at least 12 correct choices in 16 trials, over three consecutive sessions). We stopped testing if the subject did not reach the training criterion within a maximum of 10 sessions. Inter-trial intervals were kept as short as possible.

### Behavioural tests: personality

We tested 27 subjects (17 males, 10 females) using an established personality test battery [cf.^41,42^]; that is, five different tests that were conducted twice to assess temporal and contextual consistency: (i) General Activity (GA), (ii) Novel Object (NO), (iii) Novel Food (NF), (iv) Foraging Under Risk (FUR), and (v) Predator (P). All tests were conducted in a small experimental cage (see ESM and Šlipogor et al.^42^). Each experiment started with opening of the experimental cage entrance door and lasted for 300 s. The experimental set-up was placed in the furthest point of the experimental cage (diagonally to entrance) on an opaque plastic plate, and it differed based on the test: in General Activity, where monkeys were exposed to an experimental situation, the plate was empty. In Novel Object and Novel Food tests, subjects encountered a novel object or novel food, respectively, and in P test we exposed subjects to a plastic model of a snake that was hidden in leaves. In Foraging Under Risk test, we simultaneously showed subjects food rewards and a fear-evoking stimulus (i.e., a lychee fruit with skin, as established previously). For further details, refer to previous studies^41,42^. The order of subjects was randomized. All subjects participated in one test per testing day, that was always conducted in the mornings (9:00–12:00), with a three-day break between two testing days, and a two-week break between the two testing sessions in which no experiments were conducted. Water was always available.

### Video recording and coding

For SMTs, we observed and dictated all subjects’ behaviours with a voice recorder (AOSO UR28) to record the time taken to reach a particular training goal. For DLTs, we recorded subjects using one camera (Canon Legria HF G25) and video-coded the total time in seconds that an individual needed to reach criterion. For personality tests, we recorded subjects from two different angles using two video cameras (Canon Legria HF G25), merged the two videos into a single video, using a video editing software (CyberLink Power Director, version 15), and video-coded data (see^42^) using Solomon coder beta v. 17.03.22^98^. For reliability purposes we calculated inter-observer reliability for approximately 10% of the videos. For cognitive tests, the inter-observer reliability of independent coder was very high both for frequencies of behavioural variables (intra-class correlation coefficient, ICC (3,1) = 0.957, 95% CI lower, upper = 0.954, 0.960, F = 23.345, *P* < 0.001) and for durations and latencies of behavioural variables (ICC (3,1) = 0.991, 95% CI lower, upper = 0.990, 0.991, F = 105.749, *P* < 0.001). For personality tests, the inter-observer reliability of independent coder was very high for frequencies as well as for latencies and durations of behavioural variables (please see details in^42^).

### Data analysis: cognitive performance

We analysed all data with SPSS Statistics v. 23.0 (IBM). All tests were two-tailed, and we set alpha to 0.05. Learning speed (i.e., the number of seconds taken to reach criterion) was calculated for each of the five tasks and then z-transformed (i.e., standardized, so that the mean of each variable is a zero, and standard deviation is a 1.0), to improve homogeneity and comparability of the data. We labelled the z-transformed variables *‘Learning Speed’* (i.e., for ‘Target’, ‘Room’, ‘Scale’, ‘Discrimination Feature’ and ‘Discrimination Size’ separately). We then estimated consistency in learning performance across these different learning tasks, by using intra-class correlation coefficients (ICC (3,1)) and Cronbach’s Alpha. Subsequently, we summed up values for total time taken to reach criterion across the different cognitive tasks (‘Target’, ‘Room’, ‘Scale’, ‘Discrimination Feature’ and ‘Discrimination Size’), z-transformed it, and refer to it as *‘Overall Learning Speed’*. We checked whether *‘Overall Learning Speed’* was correlated with age, by using Spearman’s Rank Order Correlations, or whether it was dependent on breeding status or sex, by using Mann–Whitney U tests. To assess whether and how these learning tasks cluster, that is, whether some of them are more associated than others (i.e., not only based on our own opinion and initial division of tasks into SMTs and DLTs), we conducted a principal component analysis (PCA) on the total time needed to reach criterion, that is, we entered variables *‘Learning Speed’* of separate tasks into the PCA. The PCA-solution was Varimax-rotated, variable loadings > 0.4 and < − 0.4 were considered salient, and the components’ independence was corroborated with a direct Oblimin rotation. We further checked whether there is a consistency in learning speed within two sub-categories of learning tasks, namely we inspected it separately for SMTs (‘Target’, ‘Room’, ‘Scale’) and DLTs (‘Discrimination Feature’, ‘Discrimination Size’) using ICCs.

### Data analysis: personality

We tested for temporal consistency of behavioural variables across two test sessions and their contextual consistency (i.e., across different tests), by using ICCs (3,1). We ran a PCA with a Varimax rotation and confirmed the components with a direct Oblimin rotation. We accounted for our small sample size with running a regularized exploratory factor analysis (REFA)^99^, and the obtained REFA- and PCA-solutions were almost identical. We used eigenvalues (> 1), scree plots and Horn’s Parallel Analysis with 1000 iterations to elucidate the number of components to retain^100^. By running a bootstrapped PCA (i.e., 1000 random resamples) with a program syntax for SPSS^101^, we further confirmed the number of factors to retain, stability and replicability of the component structure (see^41^). We calculated PCA component scores with a regression method (see^42^ and ESM Table S4, for the personality structure as obtained by the PCA). We extracted four personality components, which together explained 80.84% of the variance, yet we kept only the first three components, as the PCA eigenvalues were larger than the percentiles obtained by parallel analysis^42^. The first component, ‘Exploration-Avoidance’ (36.83%), related to exploratory tendencies and stimuli manipulation. The second component, ‘Boldness-Shyness’ (19.86%), consisted of variables related to bold tendencies, staying close to, and keeping visual contact with the stimulus. The third component, ‘Stress/Activity’ (14.18%), related to both the behaviours indicative of stress as well as the animals’ locomotory patterns (for details see^42^). We used the first two personality components in subsequent analyses, namely ‘Exploration-Avoidance’ and ‘Boldness-Shyness’, as these are the personality traits usually linked with cognitive performance.

### Data analysis: cognitive performance and personality traits

We used generalized linear mixed models (GLMMs) to assess the effect of personality traits (component scores of Boldness-Shyness and Exploration-Avoidance), family group (Pooh, Sparrow, Ginevra, Veli and Kiri) and sex (female, male) on the *‘Overall Learning Speed’* across all cognitive tests, i.e., on the variable that represents the sum of their learning speed from all cognitive tests, and then separately for the SMTs and DLTs (as regression factor scores obtained by PCA, see “Results”) on the 22 subjects that were tested in both personality and cognitive test batteries. In the initial full models done for *‘Overall Learning Speed’*, and then separately for PCA-derived regression factor scores, we included family group, sex, component scores of Exploration-Avoidance and Boldness-Shyness, and the two-way interactions between group and component scores of Exploration-Avoidance and Boldness-Shyness as fixed factors. We performed a model selection to find the best-fitting models for our data. We used a backward stepwise approach based on the model comparisons of the corrected Akaike Information Criteria (AICc). We compared the model with the lowest AICc value ($\Delta$AIC = 0) with other candidate models. Following Burnham & Anderson^102^, we treated the competing models with a $\Delta$AICc < 2 as having substantial amount of support for the data and models with $\Delta$AICc < 5 as having considerably less amount of support for the data from the model with the lowest AICc value. However, we chose those models if they were less complex than the model with the lowest AICc value.

## Results

### Cognitive performance across tasks

**Figure 1:**
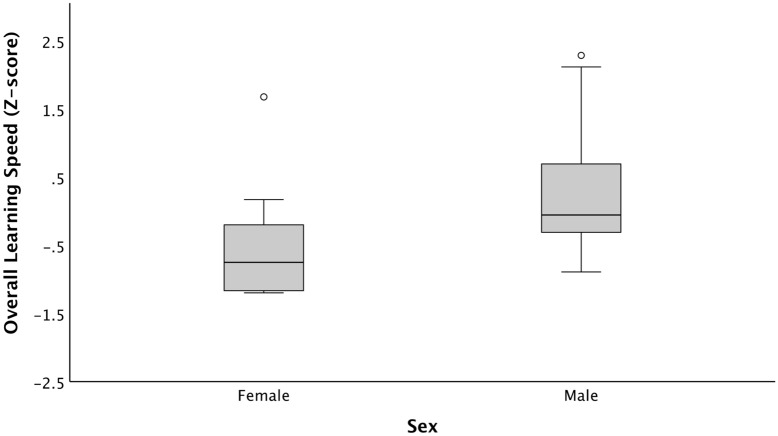
Sex differences in *Overall Learning Speed* (Z-score). *‘Overall Learning Speed* (Z-score)*’* signifies summed up values for total time taken to reach criterion across the different cognitive tasks, after a standardization, from all individuals. Low values on axis represent faster learning speed, whereas higher values represent slower learning speed. Box-plot limits indicate 25th and 75th percentiles; whiskers extend 1.5 times the interquartile range, dots indicate outliers.

We first tested whether marmosets exhibit consistent inter-individual differences across all learning tasks (i.e., ‘Target’, ‘Room’, ‘Scale’, ‘Discrimination Feature’ and ‘Discrimination Size’) using intra-class correlation coefficients (ICCs), and if the speed of learning depends on their age, sex and breeding status. We found that the ‘*Learning Speed’* was consistent across all tasks (ICC = 0.483, 95% CI lower, upper = 0.040, 0.759, F = 1.934, *P* = 0.018). We found that ‘*Overall Learning Speed’* was not correlated with age (Spearman Correlation: *r*_*s*_ = 0.04, *P* = 0.861), and did not differ between monkeys with different breeding status (breeders vs. helpers: Mann–Whitney U Test; U = 41, Z = − 1.024, *P* = 0.306). However, we found a significant sex difference in ‘*Overall Learning Speed’*; namely, females learned significantly faster than males across all learning tasks (Mann–Whitney U Test; U = 23, Z = − 2.252, *P* = 0.024; Fig. 1).

### Cognitive performance across SMTs and DLTs

To assess whether and how different learning tasks associate with each other (i.e., to corroborate the division of tasks into SMTs and DLTs), we conducted a PCA on the total time needed to reach criterion, that is, *‘Learning Speed’* (in ‘Target’, ‘Room’, ‘Scale’, ‘Discrimination Feature’ and ‘Discrimination Size’). The analyses indicated appropriate sampling adequacy (Kaiser–Meyer–Olkin measure, KMO = 0.609; Bartlett’s Test of Sphericity, *P* < 0.001), and all variables had communality estimates > 0.633 (*‘Learning Speed DS’* had the lowest communality). Components did not correlate with each other (highest factor intercorrelation: *r* = − 0.09). We extracted two principal components, which together explained 71.96% of the variance. The first component explained 48.78% of the variance and had high positive loadings (> 0.7) of *‘Learning Speed T’*, *‘Learning Speed R’* and *‘Learning Speed S’*. Thus, it consisted of variables featuring the SMTs, so we labelled the component ‘*Simple Motor Tasks: PC1’* (Table 1). The second component explained 23.19% of the variance. It had high positive loadings (> 0.7) of ‘*Learning Speed DF’* and *‘Learning Speed DS’*, and thus it consisted of variables related to the DLTs, so we labelled it ‘*Discrimination Learning Tasks: PC2’* (Table 1). Consequently, the PCA results corroborated our initial grouping of the learning tasks into simple motor tasks, and discrimination learning tasks.

**Table 1:** Variable loadings in a principal component analysis (PCA).

Variables	Components		Communalities
	Simple Motor Tasks: PC1	Discrimination Learning Tasks: PC2	
*Learning Speed T*	**0.900**		0.818
*Learning Speed R*	**0.770**		0.641
*Learning Speed S*	**0.929**		0.863
*Learning Speed DF*		**0.728**	0.642
*Learning Speed DS*		**0.774**	0.633
Eigenvalues	2.439	1.159	
% Variance	48.78	23.19	

Varimax rotation with Kaiser normalization. Loadings > 0.7 are indicated in boldface. Communalities indicate a proportion of each variable's variance that can be explained by the principal components. Eigenvalues indicate eigenvalues as obtained by the PCA. T = Target task, R = Room task, S = Scale task, DF = Discrimination Feature task, DS = Discrimination Size task.

We used the regression factor scores of these two components in further analyses. Additionally, we checked consistency in *‘Learning Speed’* separately for the different types of learning tasks. Performance in different simple motor tasks (i.e., ‘Target’, ‘Room’ and ‘Scale’) was consistent within an individual (ICC = 0.832, 95% CI lower, upper = 0.663, 0.923, F = 5.939, *P* < 0.001), whereas performance in different discrimination learning tasks (i.e., ‘Discrimination Feature’ and ‘Discrimination Size’) was not consistent within an individual (ICC = 0.276, 95% CI lower, upper = − 0.615, 0.675, F = 1.381, *P* = 0.213). Further, we looked at whether the individuals needed more time to reach criterion for different objects in the ‘Discrimination Feature’ and ‘Discrimination Size’ task. We did not find a significant difference in learning speed for the two objects used in the ‘Discrimination Feature’ task (Mann–Whitney U Test, yellow rubber duck vs. blue rubber rabbit: U = 62, Z = − 1.154, *P* = 0.249), but there was a significant difference in learning speed for the different objects used in the ‘Discrimination Size’ task (small ball vs. big ball: Mann–Whitney U Test; U = 42, Z = − 2.160, *P* = 0.031); namely, subjects assigned to a bigger object needed more time to reach the learning criterion than subjects assigned to a smaller object.

### Effect of personality and group membership on cognitive performance

**Figure 2:**
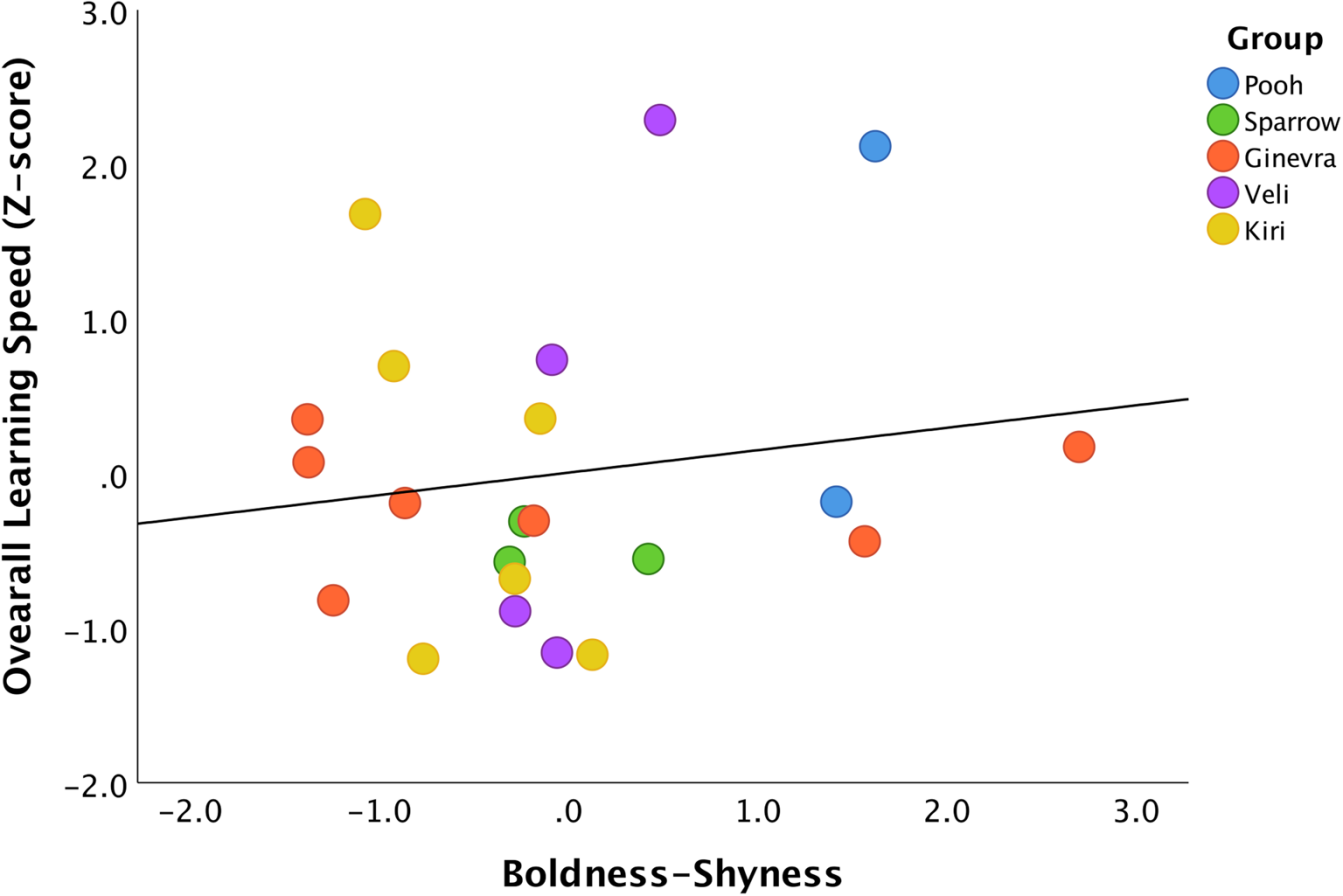
Interaction effect of Boldness-Shyness and Group Membership on *Overall Learning Speed* (Z-score). ‘*Overall Learning Speed* (Z-score)’ signifies summed up values for total time taken to reach criterion across the different cognitive tasks, after a standardization. Low values on ‘*Overall Learning Speed* (Z-score)’ axis represent faster learning speed, whereas higher values represent slower learning speed. Low values on the PCA-obtained component Boldness-Shyness represent bolder individuals, whereas higher values represent shyer individuals. Different family groups are depicted in different colours (Pooh = blue; Sparrow = green; Ginevra = orange; Veli = purple; Kiri = yellow). Black line indicates overall fit line. Every point signifies an individual.

To study the effects of personality, group membership and sex on cognitive performance, we ran GLMMs. The best fitting model selected via backward stepwise approach based on the AICc model comparisons on the *‘Overall Learning Speed’* (i.e., also better fitting than the null-model), was a model including group, sex, Boldness-Shyness and an interaction effect of group and Boldness-Shyness. In particular, Boldness-Shyness predicted cognitive performance, with bold individuals learning faster than shy individuals (Boldness-Shyness: F = 6.521, df1,2 = 1,11, *P* = 0.027, ß ± SE = -1.455 ± 0.650, t = − 2.239, 95% CI [− 2.886, − 0.025]), and there was an interaction effect of Boldness-Shyness with group membership (F = 5.438, df1,2 = 4,11, *P* = 0.012), which was driven by the groups ‘Pooh’ and ‘Veli’; these groups consisted of relatively shyer individuals that also learned more slowly (Pooh: ß ± SE = 12.643 ± 4.823, t = 2.621, 95% CI [2.027, 23.259]; Veli: ß ± SE = 5.460 ± 1.386, t = 3.940, 95% CI [2.410, 8.510]). Other effects were not significant (Fig. 2, ESM Supplementary Table S5). As the family group ‘Pooh’ consisted of only two individuals, we re-ran these analyses without them. However, as the results stayed the same, we decided to include them in our analyses. When we analysed the data across SMT and DLT separately, the best model across SMTs was a model including group, sex, Boldness-Shyness and an interaction effect of group and Boldness-Shyness. Namely, females learned significantly faster than males (Sex: F = 6.735, df1,2 = 1,11, *P* = 0.025, ß ± SE = − 0.933 ± 0.359; t = − 2.595, 95%CI [− 1.723, − 0.142]), bolder individuals learned faster than shy ones (Boldness-Shyness: F = 12.578, df1,2 = 1,11, *P* = 0.005, ß ± SE = 1.081 ± 0.651; t = 1.659, 95% CI [− 0.353, 2.514]) and there was an interaction effect of group and Boldness-Shyness (F = 4.249, df1,2 = 4,11, *P* = 0.025), where again the groups ‘Pooh’ and ‘Veli’ consisted of shyer individuals who learned more slowly (Pooh: ß ± SE = 13.081 ± 4.834, t = 2.706, 95% CI [2.441, 23.720]; Veli: ß ± SE = 2.365 ± 1.389, t = 1.703, 95% CI [− 0.692, 5.422]). The main effect of group was not significant (ESM Supplementary Table S5). The best model across DLTs included group membership, and an interaction effect of group and Boldness-Shyness (ESM Supplementary Table S5). There was a significant interaction effect of group membership and Boldness-Shyness (F = 3.108, df1,2 = 5,12, *P* = 0.05), where again the groups ‘Pooh’ and ‘Veli’ consisted of shyer individuals who learned more slowly (Pooh: ß ± SE = 6.205 ± 5.073, t = 1.223, 95% CI [− 4.848, 17.257]; Veli: ß ± SE = 2.641 ± 1.295, t = 2.039, 95% CI [− 0.181, 5.462]). The main effect of the group was not significant (ESM Supplementary Table S5).

## Discussion

Common marmosets showed substantial inter-individual consistency in how fast they reached criterion in different learning tasks. This cognitive performance across tasks was not affected by the subjects’ age and breeding status, but was affected by sex, with females learning faster than males. Furthermore, the personality trait Boldness-Shyness and the interaction between Boldness-Shyness and group membership, best explained the variation in learning speed across all learning-focused cognitive tasks.

That common marmosets showed inter-individual consistency in learning speed, and thus can be classified along an axis of fast-slow learners, is in line with previous results from other species^17^ and fits to genus-level differences in neophilia and innovation reported for Callithrichid monkeys^78^. The effect was particularly evident across those tasks that featured simple motor problems like holding a target (i.e., ‘Target’ task) or sitting on a scale while holding a target (i.e., ‘Scale’ task). In the two discrimination tasks, however, the pattern was less clear and likely affected by a motivational confound in the size discrimination (DS) task. Note that in this task, individuals that had to associate the large object with the reward, needed considerably more time to differentiate between the objects, possibly because they had initial problems to approach and/or touch the large object. These monkeys’ performance in the DS task might thus reflect some initial neophobic response or fear/aversion, despite our attempts to control for such effects, by pre-habituating subjects to the objects and boards in DLTs (that were placed in front of the subjects’ home cages for five days before the tests started), and by ‘biasing’ them additionally for their (S+) object in the pre-session. Inconsistency in the individuals’ performance across learning tasks has recently been described also for other species like guinea pigs, *Cavia aperea f. porcellus*^14^ and mouse lemurs^56^, and linked to motivational confounds, preferences for particular objects, colours or shapes, environmental factors and/or previous experiences^3,11,58^.

Although our test subjects covered nearly the entire age spectrum of marmosets, from 0.5 years to 15 years, we did not find any age effect on learning speed. These results are in line with previous literature on visual shape discrimination in common marmosets^103,104^ and other primate species (e.g.,^105–107^), but stand in contrast to some recent literature indicating a cognitive decline in marmosets when tested in reversal learning tasks^108^. Possibly this difference is due to the additional cognitive skills required for reversal learning, like the inhibition of a learned response and flexibility in reversing the reward contingencies^109^ that may be further related to challenges faced in the social or foraging domain^110,111^. Learning speed did not differ between breeders and helpers, but, contrary to our expectation, it differed between males and females. The latter is particularly interesting as sex effects on learning performance have been found in some^108,112,113^, but not all studies on marmosets^81,103,104^. That our males were learning more slowly than females fits to recent findings that marmoset males have slightly longer response times than marmoset females for randomized delays^108^, are more prone to distraction and demotivation, and are more interested in the environment and less likely to work for food than females^112,113^. Our findings are also supported by framework laid out by Yamamoto et al.^114^ who argued that breeding marmoset females should learn more quickly in food-related tasks, due to their higher energetic demands connected with reproduction.

As expected, we found the predicted link between learning and personality^22^, whereby sex-specific and social factors also came into play. Specifically, the personality trait Boldness-Shyness, group membership and sex, together with the interaction of Boldness-Shyness and group membership, predicted the marmosets’ learning speed across all tasks. Overall, bolder individuals learned faster than shy individuals, and shy individuals belonging to some family groups learned particularly slowly. When we analysed the two types of learning tasks separately, the overall pattern held for simple motor tasks (SMTs), that is, females learned considerably faster than males, bolder individuals learned faster than shy ones and shy individuals belonging to some groups learned more slowly than others. In the discrimination learning tasks (DLTs), learning speed was predicted by group membership and interaction between group membership and Boldness-Shyness. Hence, shyer individuals belonging to some groups learned more slowly than other individuals. Nevertheless, one group (i.e., ‘Kiri’) showed an opposite pattern, namely that shy individuals learned faster. One might argue that in one task (‘Room’), the learning-aspect of the test may have been convoluted with personality: bolder individuals may have simply been faster in exploring all parts of the test cage, and we may have measured their personality rather than learning. However, we accounted for this by providing animals with food rewards for desired behavioural outcome, which would not have been used in a standard personality test. We also corroborated our findings by repeating the analyses without the ‘Room’ task and our results remained largely the same.

These results suggest that both personality and social environment (i.e., group membership) are important factors for explaining learning speed in common marmosets, across a variety of learning tasks, and the proximate basis of this behaviour as well as its maintenance should be explored in future studies. Yet, as this variability may have been driven by a few outliers and the relatively small sample size overall, we need to treat these results with caution. Differences in learning speed among family groups could indicate possible effects of shared social environment and previous experiences, but also of shared genetics^40^; together, these factors may shape the direction and strength of the links between personality and cognitive performance in cooperatively breeding primates and possibly other mammalian and avian taxa with a similar social structure.

Our key finding that bolder marmosets are faster learners supports the framework proposed by Sih & Del Giudice^22^, where bold, more aggressive, and/or less social personality types should prioritize learning speed over accuracy; bold individuals should thus be fast in learning new tasks that require high levels of activity, or interactions with novel set-ups. Notably, our simple motor tasks perfectly fit this prediction both in design and outcome. Complementary to our results, marmosets with higher emotional reactivity have lower participation, but not a lower performance in a non-associatively learned cognitive task (i.e., finding food using human cues^113^). While these studies on marmosets are in line with Sih & Del Giudice’s model^22^, a recent meta-analysis failed to find that bold or proactive individuals are consistently faster learners^18^, possibly because factors like the social environment and previous experience have not been accounted for in some studies (see also^11^). Indeed, the current overall picture on the interplay between personality, social environment and cognitive performance is far from clear, and seems to depend on the species and given context^14,115^. In evolutionary terms, a positive relationship between boldness, cognitive performance and social environment is plausible, as bolder individuals and species tend to disperse more^116^, and thus experience and act upon new situations, food, or physical and social environments. Particularly the latter could promote individualized social relationships^117^ and select for higher socio-cognitive performance^64^ in not only these phenotypes, but also their groups or populations.

Taken together, we found that common marmosets can be classified along the axis of fast-slow learners, whereby the slow learners of our colony tended to be shy and clustered in some family groups. It remains to be explored whether these findings hold with other, possibly more complex, cognitive tasks (e.g., reversal learning, social learning, inferential reasoning) and under ecologically relevant field conditions. Further studies linking inter-individual behavioural and cognitive variation with social environment are much needed to understand the generalizability of the described phenomena, not only in marmosets but in various socially living species.

## Supplementary Information

Supplementary Information 1.

Supplementary Information 2.

## Data Availability

The datasets supporting this article have been uploaded as part of the Electronic Supplementary Material.
