# Supplementary material for: Personality and social environment predict cognitive performance in common marmosets (Callithrix jacchus)
Source: Sci Rep. 2022 May 5;12:6702. doi: 10.1038/s41598-022-10296-8 (PMC9072541; doi:10.1038/s41598-022-10296-8)
Supplement: Supplementary file 2 — Supplementary Information 2. [file 41598_2022_10296_MOESM2_ESM.pdf]

<u>**Electronic Supplemental Material:**</u>

Personality and Social Environment Predict Cognitive Performance in Common Marmosets (*Callithrix jacchus*)

Šlipogor, V.*, Graf, C., Massen, J. J. M., & Bugnyar, T.

<u>*author for correspondence</u>

INDEX

Supplementary Methods 2

Housing Conditions 2

Table S1. Description of the 'Target' task 3

Figure S1. 'Target' task 4

Table S2. Description of the 'Room' task 5

Figure S2. 'Room' task 6

Table S3. Description of the 'Scale' task 7

Figure S3. 'Scale' task 8

Figure S4. Discrimination Learning Tasks 9

Table S4. Personality Structure as obtained with PCA 10

Table S5. Best-Fitting Models (GLMMs) of Cognitive Performance 11

Supplementary Methods

Housing Conditions. The monkeys were kept in two rooms and housed in indoor-outdoor wire mesh enclosures (approximately 500 cm x 250 cm x 250 cm) in five family groups, that were visually isolated from each other, but maintaining acoustic and olfactory contact. During the time of testing, one family group ("Ginevra") was kept in two sub-groups for management reasons, but they could freely interact through the mesh. Enclosures were equipped with natural tree branches, bamboos, wooden boards, baskets, climbing and tunnel structures, hammocks, towels and other enrichment objects. Floors in indoor enclosures were covered with coniferous pellet bedding. The temperature and humidity in indoor enclosures were kept between 21-29 °C and between 30-60%, respectively. Marmosets had *ad libitum* access to water, and the food was served twice per day (at ±7 am and ±12 am). The well-balanced food included nutritive New World monkey pellets containing vitamins and minerals, various fruits and vegetables, eggs, oatmeal, nuts, insects, marmoset gum and jelly. As enrichment, monkeys got foraging boxes with meal worms or crickets, and occasionally tea or frozen fruit pulp. As additional rewards in husbandry or training procedures, monkeys received bananas, banana pellets, rice waffles, honey, blueberries or green beans, and food rewards in tests described in the manuscript were based on monkeys' individual preferences. The keeping rooms, the experimental rooms and the hallway had windows for natural light, but additional artificial lights were available, where the day:night cycle was set to 12:12 hours. Further, all indoor enclosures had infrared lamps to ensure subjects' well-being. Indoor and outdoor enclosures were connected with a passageway tunnel system to the experimental cages and to the experimental room, and the tunnels were used to guide monkeys to the different enclosures.

Table S1. Description of the 'Target' task, indicating different training phases, plans, goals, criteria, number of sessions to completion, and set-up (i.e., individual or group).

Phase	Training Plan	Training Goal	Training Criterion	Sessions	Set-up
Pre	Rewards are shown to subject 10 times in a row. Experimenter clicks and rewards as soon as the subject shows interest in the reward.	Habituation* to the experimenter holding a clicker; association of the click with a food reward.	10/10 rewards within 5 minutes	1	Individual
1	The target is held out of subject's reach. Experimenter clicks and rewards when a subject shows interest in experimenter or target (i.e., looks, sits close or moves towards experimenter or target).	Habituation* to the experimenter holding a target stick and clicker; association of experimenter or target with a click and reward (i.e., looking, sitting close or moving towards experimenter or target)	7/10 rewards within 5 minutes	5	Individual
2	Experimenter inserts the target into the experimental cage within the subject's reach. Experimenter clicks and rewards when a subject shows interest in the target (i.e., looks at or touches the target).	Habituation* to the target inside of the experimental cage; association of target stick with a click and reward (i.e., looking at or touching the target)	7/10 rewards within 5 minutes	5	Individual
3	Experimenter inserts the target into the experimental cage within the subject's reach. Experimenter clicks and rewards when a subject holds the ball-point of the target with one or both hands for an extended period (approx. 3 seconds).	Habituation* to the target inside of the experimental cage; association of holding the ball-point of the target with one or both hands for an extended period (approx. 3 seconds) with a click and reward	7/10 rewards within 5 minutes	5	Individual

*subject(s) shows no visible signs of any behaviour indicative of stress or fear

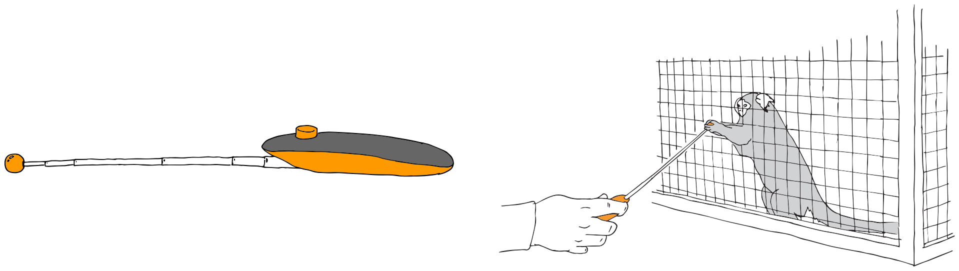

a) b)

Figure S1. 'Target' task. a) Target stick with a ball-point 'target' on top and an integrated clicker, b) a subject touching the 'target' during a session. (Figure by Mauro Milli).

Table S2. Description of the 'Room' task, indicating different training phases, plans, goals, criteria, number of sessions to completion and set-up (i.e., individual or group).

Phase	Training Plan	Training Goal	Training Criterion	Sessions	Set-up
Pre	Whole hallway tunnel system is accessible; the experimenter provides food rewards in front of the closed entrance to the test room.	Habituation* to the whole hallway tunnel system.	Reach the furthest point in the whole tunnel system.	1	Group
1	The entrance to test room is opened; the experimenter provides food rewards after subjects enter the first tunnel in the test room.	Habituation* to entering test room and to the first tunnel in test room.	Reach the first tunnel in the test room within 5 minutes.	2	Group
2	The entrance to test cage is opened; the experimenter provides food rewards in the first enclosure.	Habituation* to the first enclosure of the test cage.	Reach the first enclosure in the test cage within 5 minutes.	2	Group
3	The entrance to test cage is opened; the experimenter provides food rewards in the second enclosure.	Habituation* to the second enclosure of the test cage.	Reach the second enclosure in the test cage within 5 minutes.	2	Group
4	The entrance to all parts of the test cage is available; the experimenter provides food rewards in the furthest part of the cage.	Habituation* to the whole test cage.	Reach the third enclosure in the test cage within 5 minutes.	2	Group
5	The family group is in the first compartment; while the individual monkeys consecutively get access to all parts of the test cage; the experimenter provides food rewards in the furthest part of the cage.	Habituation* to the whole test cage while being away from the family group.	Reach the third enclosure in the test cage within 5 minutes.	5	Individual

* subject(s) shows no visible signs of any behaviour indicative of stress or fear

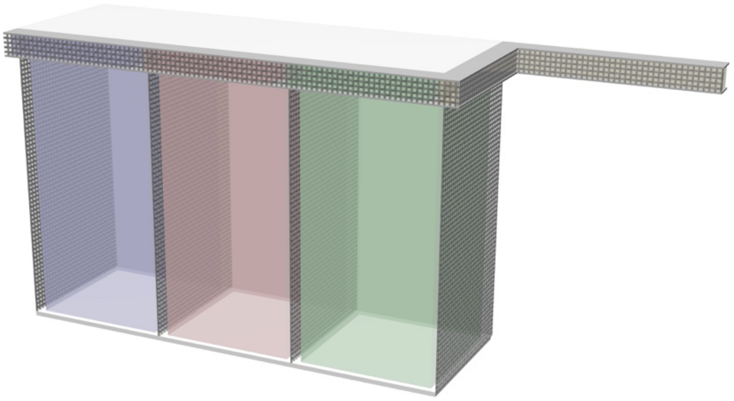

Figure S2. ‘Room’ task. Test cage with passageway tunnel system that allows subjects access to all compartments. Different training phases are highlighted in different colours (Phase 1: yellow, Phase 2: green, Phase 3: red, Phase 4: blue, Phases 1-4: group set-up; Phase 5: blue, individual set-up). (Figure by Mauro Milli).

Table S3. Description of the 'Scale' task, indicating different training phases, plans, goals, criteria, number of sessions to completion and set-up (i.e., individual or group).

Phase	Training Plan	Training Goal	Training Criterion	Sessions	Set-up
Pre	10 rewards per individual are offered on the scale and subjects can freely take them.	Habituation* to the scale and to the experimenter holding a target stick with clicker.	All subjects are coming to the scale and taking rewards.	1	Group
1	Experimenter clicks and rewards when a subject interacts with the scale (i.e., looks, touches or sits on it).	Habituation* and showing visible interest to the scale (i.e., looking, touching or sitting on the scale); association of the interaction with the scale with the click and reward.	All subjects are showing interest in the scale, no upper reward limit per individual within 5 minutes	2	Group
2	Experimenter inserts the target into the experimental cage within the subject's reach. Experimenter clicks and rewards when a subject is sitting on the scale and touching the target with one or both hands (approx. 1 second).	Habituation* to and sitting on the scale while touching the target with one or both hands; association of touching the target with one or both hands while sitting on a scale (approx. 1 second) with the click.	7/10 rewards within 5 minutes	5	Individual
3	Experimenter inserts the target into the experimental cage within the subject's reach. Experimenter clicks and rewards when a subject is sitting on the scale and touching the target with one or both hands for a prolonged period (approx. 3 seconds).	Habituation* to and sitting on the scale while touching the target with one or both hands for a prolonged period (approx. 3 seconds); association of touching the target with one or both hands while sitting on a scale for a prolonged period with the click.	7/10 rewards within 5 minutes	5	Individual

* subject(s) shows no visible signs of any behaviour indicative of stress or fear

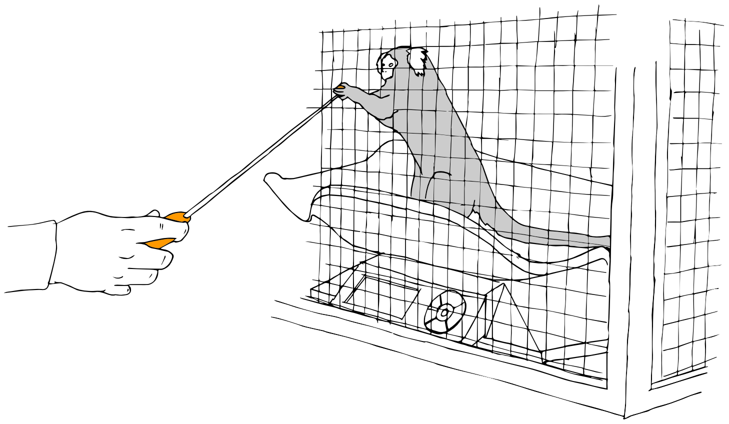

Figure S3. ‘Scale’ task. A subject is standing on a scale while touching a ball-point ‘target’ of the target stick with both hands. (Figure by Mauro Milli).

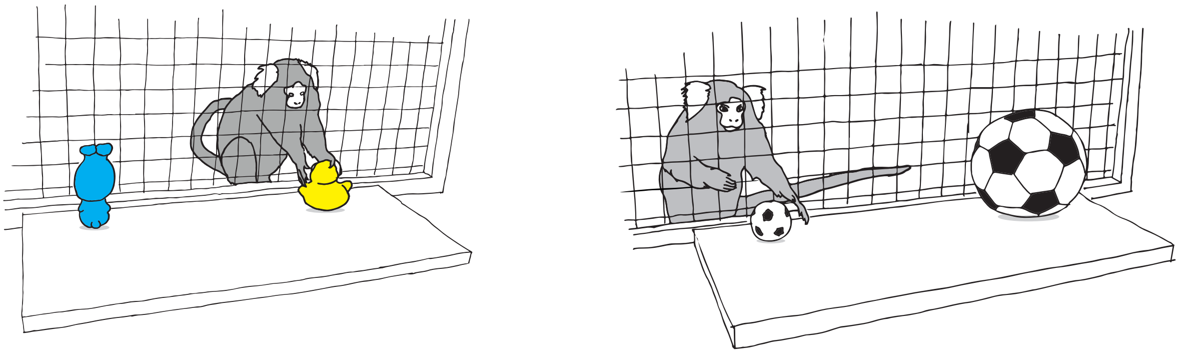

a) b)

Figure S4. Discrimination Learning Tasks: a) a subject performing a ‘Discrimination Feature’ task and b) a subject performing a ‘Discrimination Size’ task. (Figure by Mauro Milli).

Table S4. Personality Structure as obtained with a PCA. Loadings and communalities of behavioral variables in a PCA. Varimax rotation with Kaiser normalization. Variable loadings> ±0.4 are indicated in the table. Communalities are a proportion of each variable's variance that can be explained by the principal components. Eigenvalues larger than percentiles are indicated with an asterisk. GA: General Activity, NO: Novel Object, NF: Novel Food, FUR: Foraging Under Risk, P: Predator. Frequencies are noted with the letter "F", durations with the letter "D", and latencies with the letter "L" in superscript. [Adapted from Table 3, Šlipogor et al. 2021].

Component	% Variance	Eigenvalue	Behavioral Variable	Loadings	Communalities
Exploration-Avoidance	36.83	5.524*	Ground^{D} (GA, NO, FUR)<br>Manipulation Target^{D} (FUR)<br>Proximity^{D} (GA, NO, NF, FUR)<br>Food Calls^{F} (FUR)<br>Body^{L} (GA, NO, NF, FUR, P)<br>Touch^{L} (GA, NF, FUR)	0.934<br>0.827<br>0.754<br>0.710<br>-0.700<br>-0.894	0.913<br>0.778<br>0.840<br>0.544<br>0.853<br>0.895
Boldness-Shyness	19.86	2.978	Enter^{L} (GA, NO, NF, FUR, P)<br>Distance^{D} (GA, NO, NF, FUR, P)<br>Body^{L} (GA, NO, NF, FUR, P)<br>Proximity^{D} (GA, NO, NF, FUR)<br>Focus^{D} (GA, NO, NF, FUR, P)	0.847<br>0.815<br>0.578<br>-0.492<br>-0.927	0.842<br>0.806<br>0.853<br>0.840<br>0.906
Stress/Activity	14.18	2.127	Locomotion^{D} (GA, NO, NF, FUR, P)<br>Compartment Alternations^{F} (GA, NO, NF, FUR, P)<br>Stress Behavior^{F} (NO, NF, P)<br>Vigilance Calls^{F} (GA, NO, P)	0.871<br>0.854<br>0.761<br>0.716	0.906<br>0.821<br>0.598<br>0.733
Fourth	9.98	1.496	Manipulation Target^{D} (NF)<br>Contact Calls^{F} (NO, FUR, P)	0.926<br>0.900	0.864<br>0.826

Table S5. Best-Fitting Models (GLMMs) of Cognitive Performance performed on: i) *Overall Learning Speed*, ii) *Simple Motor Tasks: PC1*, iii) *Discrimination Learning Tasks: PC2*. Significant effects are indicated in bold. Reference groups are indicated in parenthesis.

target	corrected model	fixed effects	levels of fixed effects	F	(df1, df2)	β-coefficient	± SE	t	95% CI (lower, upper)	P-value
Overall Learning Speed	**group, sex, Boldness, group*Boldness**			**3.250**	**(10,11)**					**0.033**
		group (Kiri)		1.738	(4,11)					0.212
			Pooh			-15.698	7.293	-2.152	-31.751, 0.355	
			Sparrow			0.056	0.612	0.092	-1.290, 1.403	
			Ginevra			0.375	0.529	0.708	-0.790, 1.540	
			Veli			0.696	0.580	1.199	-0.582, 1.973	
		sex (Male)		3.887	(1,11)					0.074
			Female			-0.707	0.359	-1.972	-1.496, 0.082	
		**Boldness**		**6.521**	**(1,11)**					**0.027**
			Boldness			-1.455	0.650	-2.239	-2.886, -0.025	
		**group*Boldness**		**5.438**	**(4,11)**					**0.012**
			Pooh*Boldness			12.643	4.823	2.621	2.027, 23.259	
			Sparrow*Boldness			0.738	1.416	0.521	-2.379, 3.856	
			Ginevra*Boldness			1.550	0.673	2.302	0.068, 3.032	
			Veli*Boldness			5.460	1.386	3.940	2.410, 8.510	
Simple Motor Tasks: PC1	**group, sex, Boldness, group*Boldness**			**3.231**	**(10,11)**					**0.034**
		group (Kiri)		2.916	(4, 11)					0.072
			Pooh			-22.087	7.309	-3.022	-38.174, -5.999	
			Sparrow			-0.293	0.613	-0.478	-1.643, 1.056	
			Ginevra			-0.122	0.531	-0.231	-1.290, 1.046	
			Veli			-0.783	0.581	-1.346	-2.062, 0.497	
		**sex (Male)**		**6.735**	**(1,11)**					**0.025**
			**Female**			-0.933	0.359	-2.595	-1.723, -0.142	
		**Boldness**		**12.578**	**(1,11)**					**0.005**
			Boldness			1.081	0.651	1.659	-0.353, 2.514	
		**group*Boldness**		**4.249**	**(4,11)**					**0.025**
			Pooh*Boldness			13.081	4.834	2.706	2.441, 23.720	
			Sparrow*Boldness			-1.767	1.419	-1.245	-4.891, 1.358	
			Ginevra*Boldness			-0.828	0.675	-1.227	-2.313, 0.657	
			Veli*Boldness			2.365	1.389	1.703	-0.692, 5.422	
Discrimination Learning Tasks: PC2	**group, group*Boldness**			**2.957**	**(9,12)**					**0.042**
		group (Kiri)		2.342	(4,12)					0.114
			Pooh			-7.519	7.736	-0.972	-24.375, 9.338	
			Sparrow			0.226	0.632	0.358	-1.151, 1.604	
			Ginevra			0.522	0.542	0.963	-0.659, 1.703	
			Veli			1.486	0.593	2.506	0.194, 2.779	
		**group*Boldness**		**3.108**	**(5,12)**					**0.050**
			Pooh*Boldness			6.205	5.073	1.223	-4.848, 17.257	
			Sparrow*Boldness			-0.291	1.294	-0.225	-3.110, 2.527	
			Ginevra*Boldness			-0.070	0.186	-0.379	-0.475, 0.334	
			Veli*Boldness			2.641	1.295	2.039	-0.181, 5.462	
			Kiri*Boldness			-2.147	0.690	-3.113	-3.650, -0.645a	
